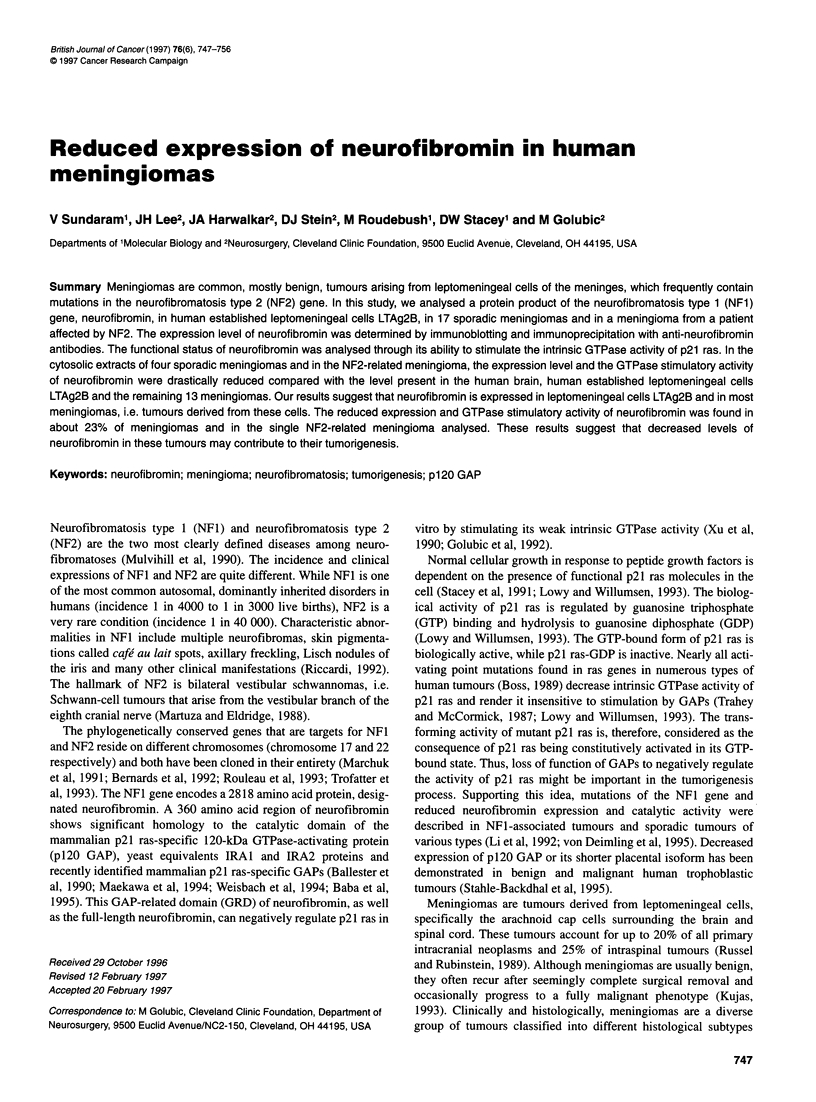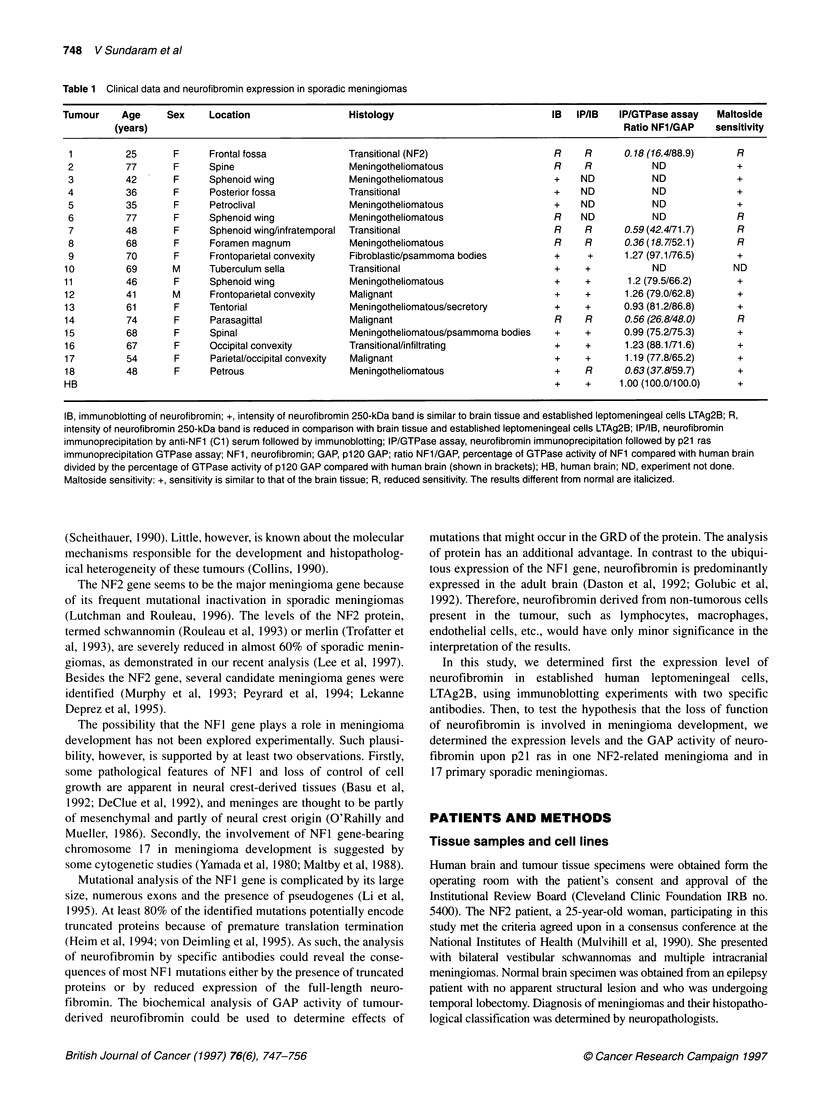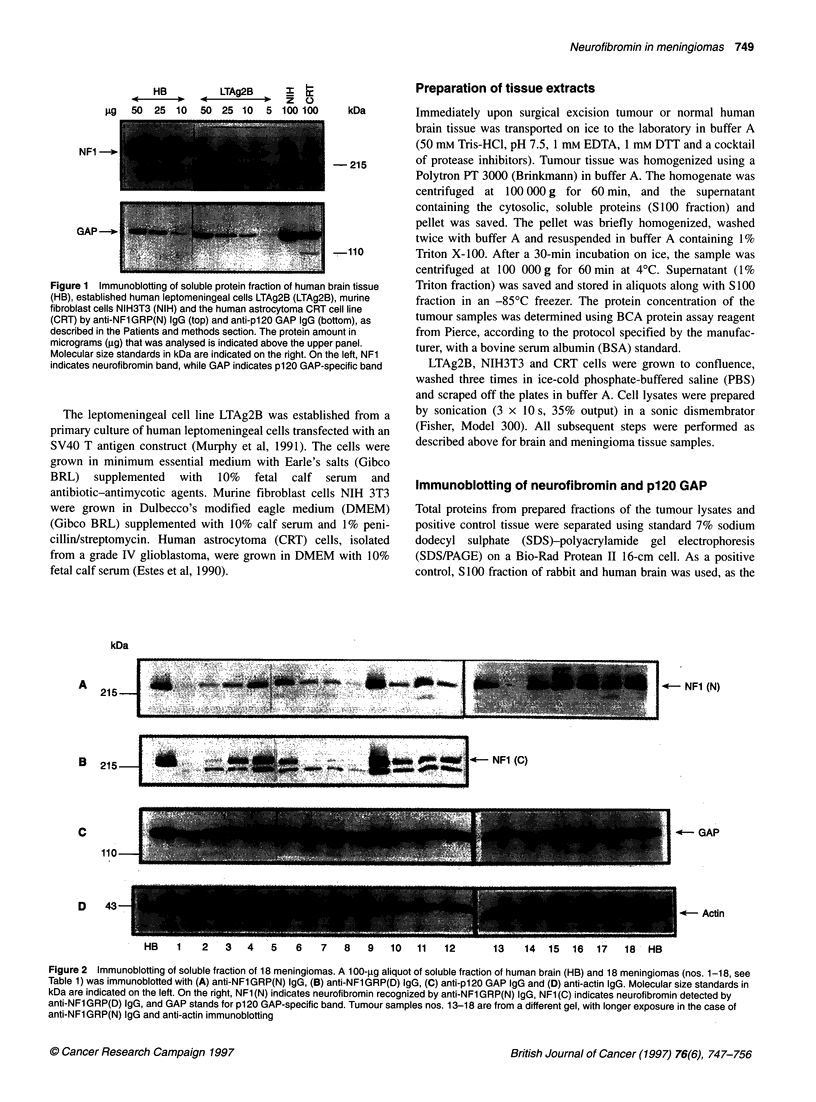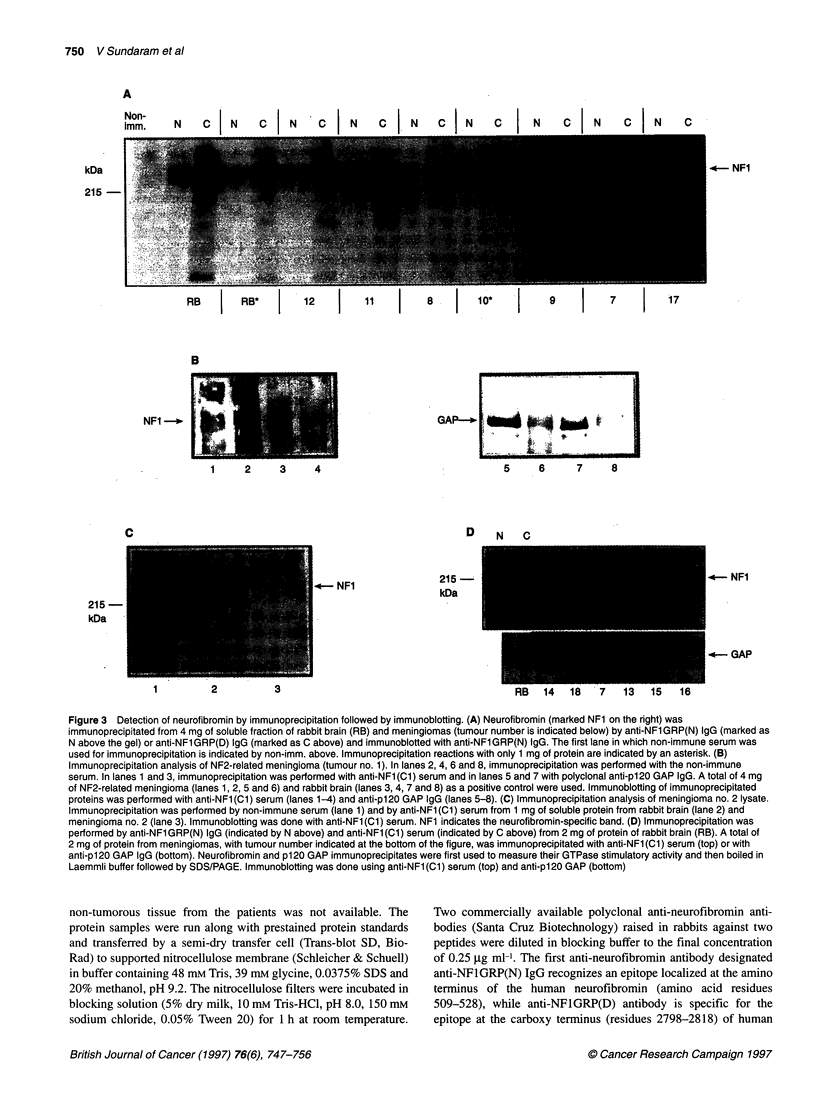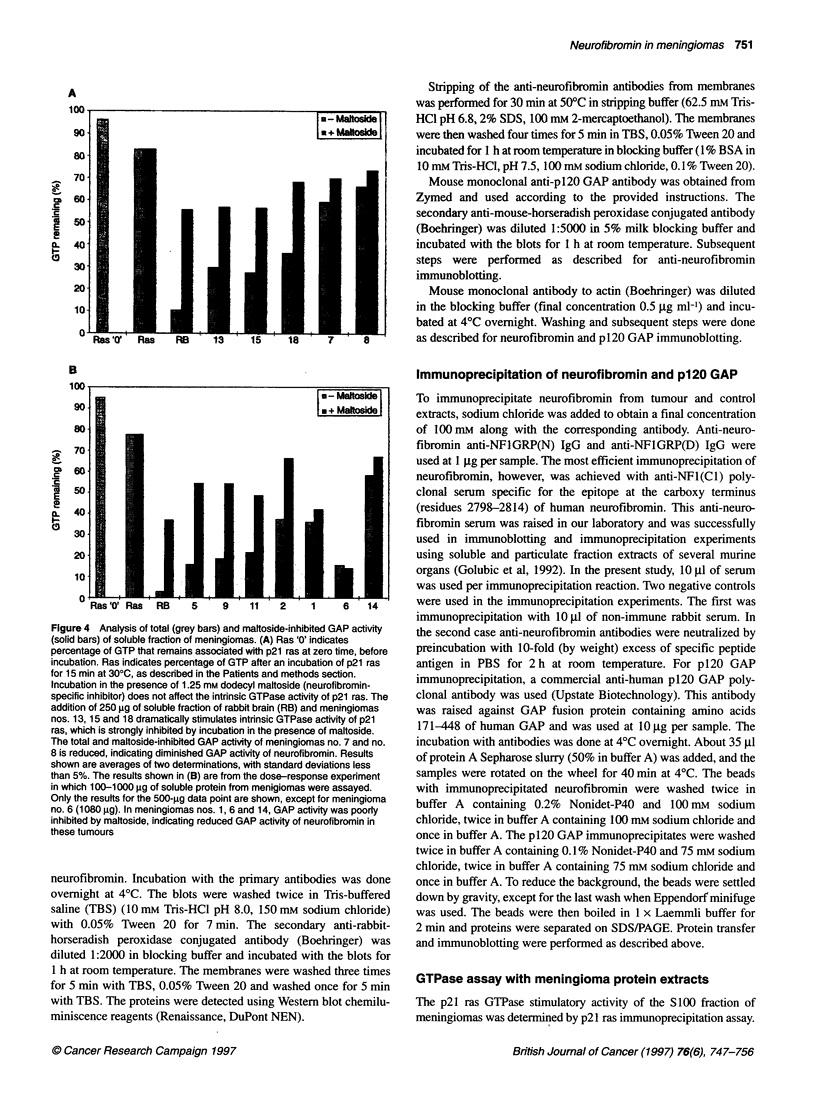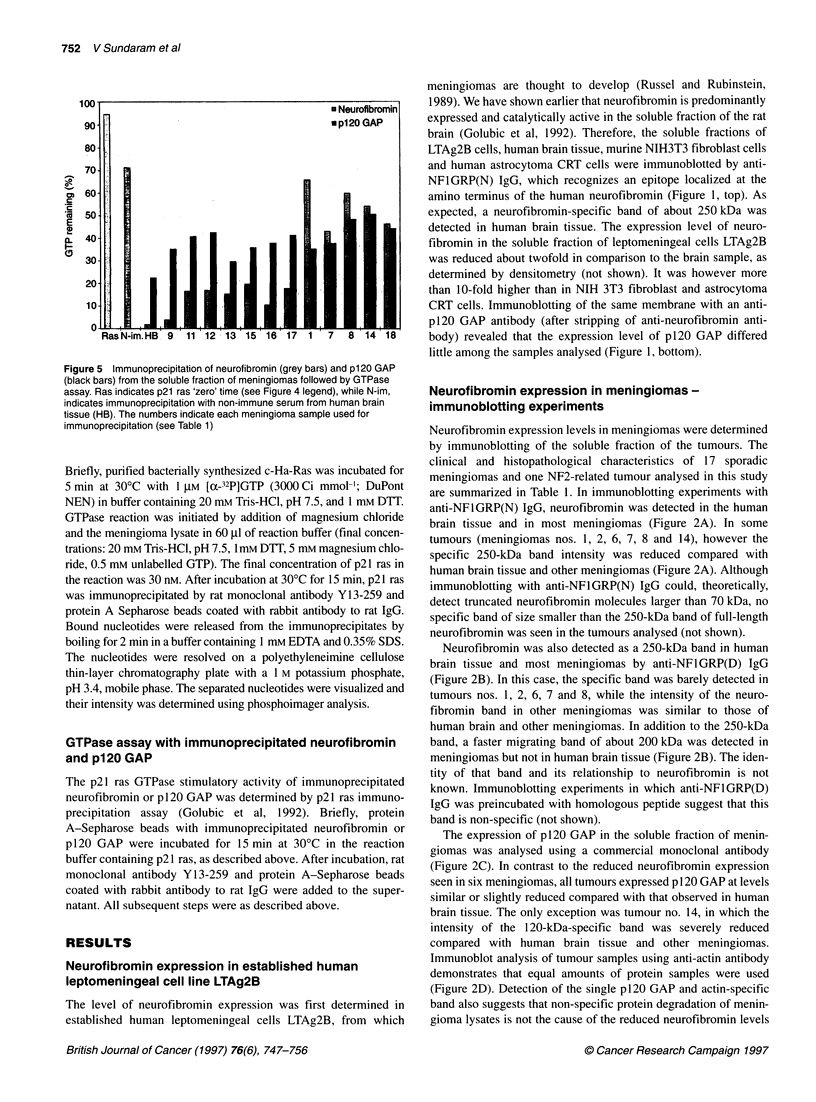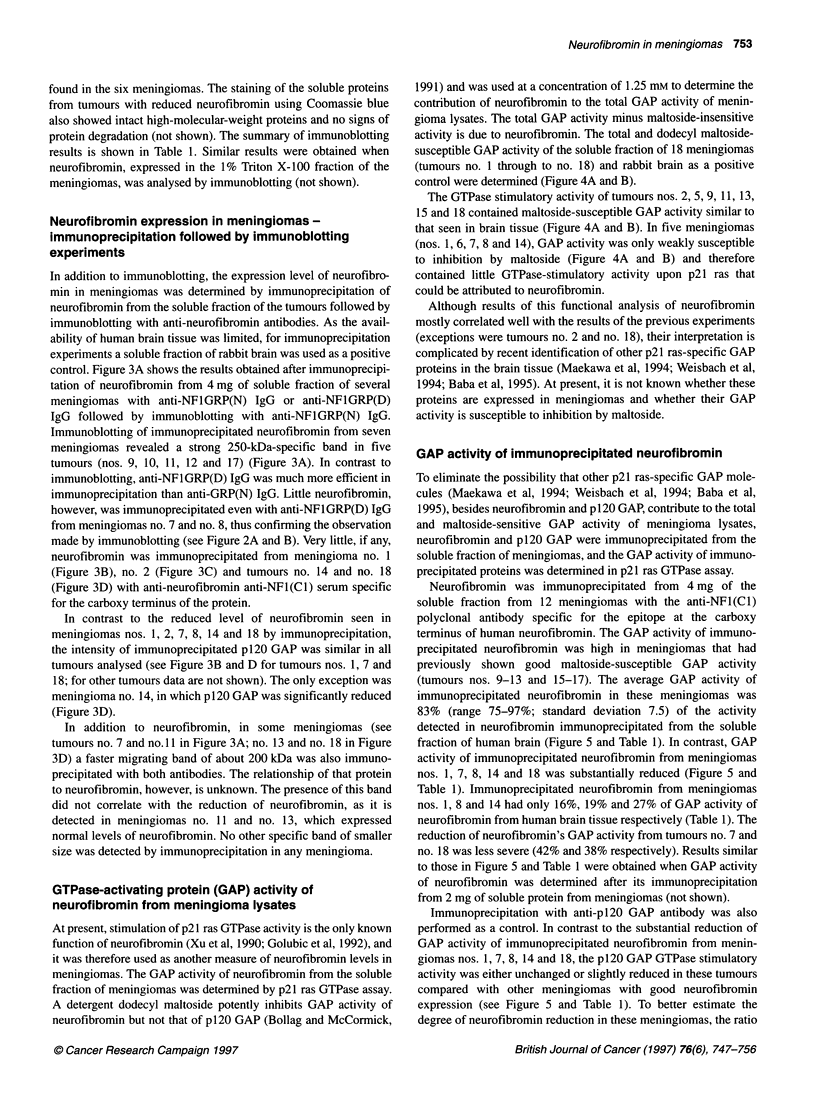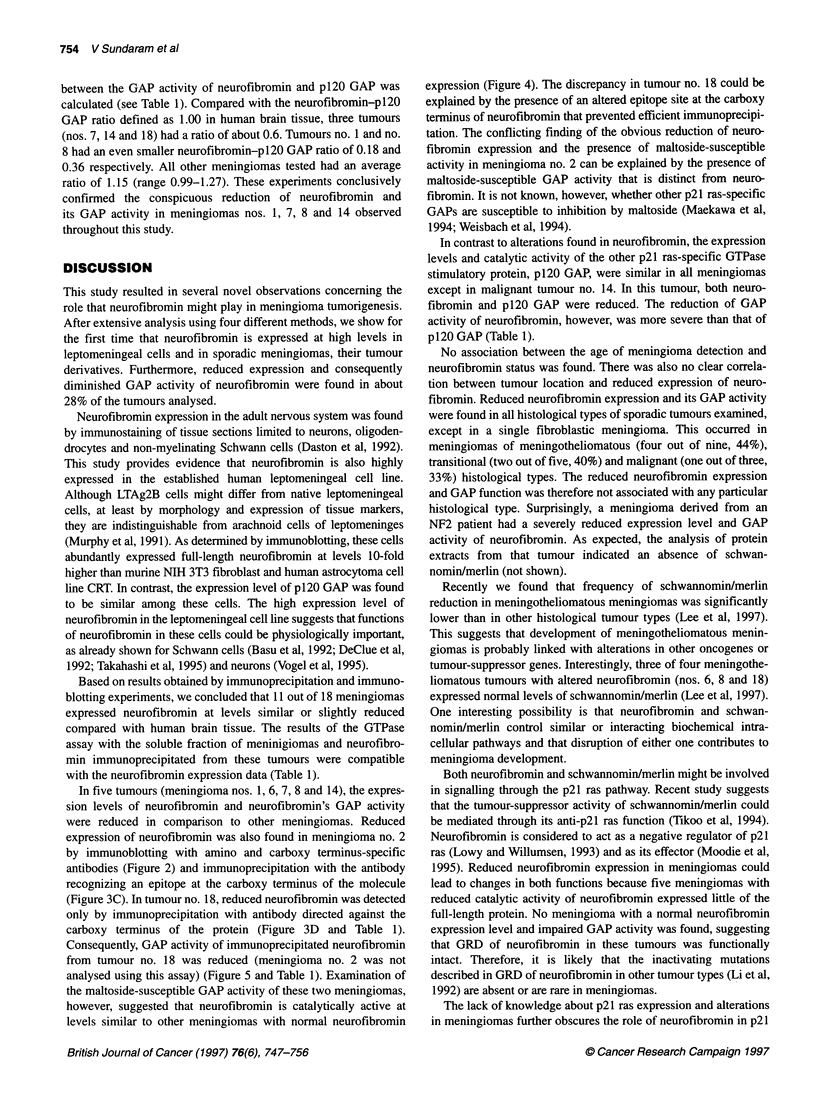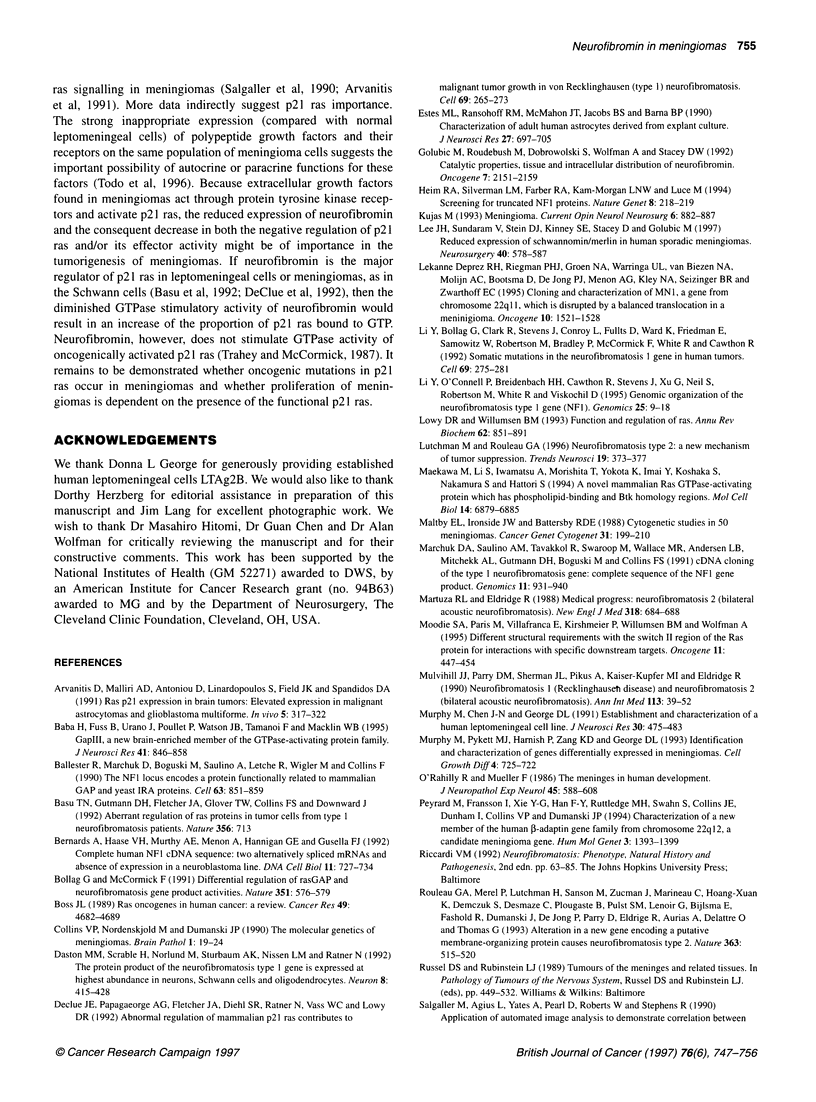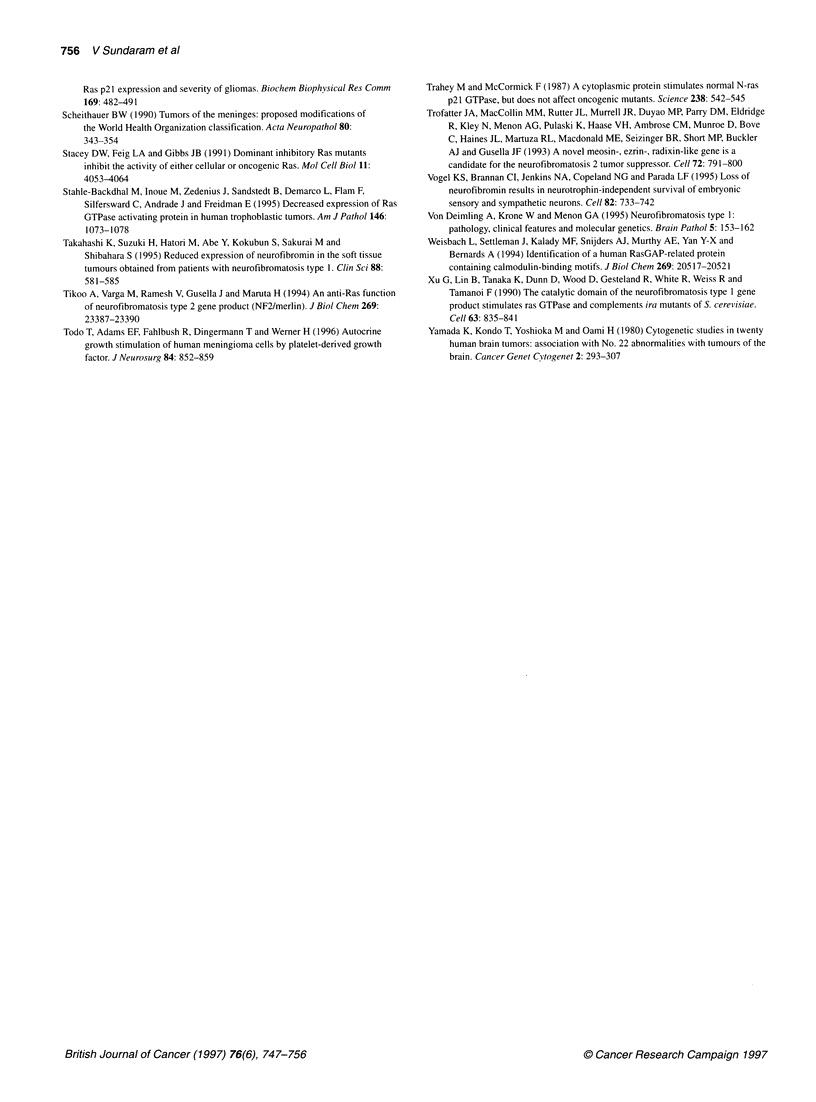# Reduced expression of neurofibromin in human meningiomas.

**DOI:** 10.1038/bjc.1997.456

**Published:** 1997

**Authors:** V. Sundaram, J. H. Lee, J. A. Harwalkar, D. J. Stein, M. Roudebush, D. W. Stacey, M. Golubic

**Affiliations:** Department of Molecular Biology, Cleveland Clinic Foundation, OH 44195, USA.

## Abstract

**Images:**


British Journal of Cancer (1997) 76(6), 747–756
© 1997 Cancer Research Campaign

### Reduced expression of neurofibromin in human meningiomas

**V Sundaram[1], JH Lee[2], JA Harwalkar[2], DJ Stein[2], M Roudebush[1], DW Stacey[1] and M Golubic[2]**

Departments of [1]Molecular Biology and [2]Neurosurgery, Cleveland Clinic Foundation, 9500 Euclid Avenue, Cleveland, OH 44195, USA

**Summary** Meningiomas are common, mostly benign, tumours arising from leptomeningeal cells of the meninges, which frequently contain mutations in the neurofibromatosis type 2 (NF2) gene. In this study, we analysed a protein product of the neurofibromatosis type 1 (NF1) gene, neurofibromin, in human established leptomeningeal cells LTAg2B, in 17 sporadic meningiomas and in a meningioma from a patient affected by NF2. The expression level of neurofibromin was determined by immunoblotting and immunoprecipitation with anti-neurofibromin antibodies. The functional status of neurofibromin was analysed through its ability to stimulate the intrinsic GTPase activity of p21 ras. In the cytosolic extracts of four sporadic meningiomas and in the NF2-related meningioma, the expression level and the GTPase stimulatory activity of neurofibromin were drastically reduced compared with the level present in the human brain, human established leptomeningeal cells LTAg2B and the remaining 13 meningiomas. Our results suggest that neurofibromin is expressed in leptomeningeal cells LTAg2B and in most meningiomas, i.e. tumours derived from these cells. The reduced expression and GTPase stimulatory activity of neurofibromin was found in about 23% of meningiomas and in the single NF2-related meningioma analysed. These results suggest that decreased levels of neurofibromin in these tumours may contribute to their tumorigenesis.

**Keywords:** neurofibromin; meningioma; neurofibromatosis; tumorigenesis; p120 GAP

Neurofibromatosis type 1 (NF1) and neurofibromatosis type 2 (NF2) are the two most clearly defined diseases among neurofibromatoses (Mulvihill et al, 1990). The incidence and clinical expressions of NF1 and NF2 are quite different. While NF1 is one of the most common autosomal, dominantly inherited disorders in humans (incidence 1 in 4000 to 1 in 3000 live births), NF2 is a very rare condition (incidence 1 in 40 000). Characteristic abnormalities in NF1 include multiple neurofibromas, skin pigmentations called *café au lait* spots, axillary freckling, Lisch nodules of the iris and many other clinical manifestations (Riccardi, 1992). The hallmark of NF2 is bilateral vestibular schwannomas, i.e. Schwann-cell tumours that arise from the vestibular branch of the eighth cranial nerve (Martuza and Eldridge, 1988).

The phylogenetically conserved genes that are targets for NF1 and NF2 reside on different chromosomes (chromosome 17 and 22 respectively) and both have been cloned in their entirety (Marchuk et al, 1991; Bernards et al, 1992; Rouleau et al, 1993; Trofatter et al, 1993). The NF1 gene encodes a 2818 amino acid protein, designated neurofibromin. A 360 amino acid region of neurofibromin shows significant homology to the catalytic domain of the mammalian p21 ras-specific 120-kDa GTPase-activating protein (p120 GAP), yeast equivalents IRA1 and IRA2 proteins and recently identified mammalian p21 ras-specific GAPs (Ballester et al, 1990; Maekawa et al, 1994; Weisbach et al, 1994; Baba et al, 1995). This GAP-related domain (GRD) of neurofibromin, as well as the full-length neurofibromin, can negatively regulate p21 ras in vitro by stimulating its weak intrinsic GTPase activity (Xu et al, 1990; Golubic et al, 1992).

Normal cellular growth in response to peptide growth factors is dependent on the presence of functional p21 ras molecules in the cell (Stacey et al, 1991; Lowy and Willumsen, 1993). The biological activity of p21 ras is regulated by guanosine triphosphate (GTP) binding and hydrolysis to guanosine diphosphate (GDP) (Lowy and Willumsen, 1993). The GTP-bound form of p21 ras is biologically active, while p21 ras-GDP is inactive. Nearly all activating point mutations found in ras genes in numerous types of human tumours (Boss, 1989) decrease intrinsic GTPase activity of p21 ras and render it insensitive to stimulation by GAPs (Trahey and McCormick, 1987; Lowy and Willumsen, 1993). The transforming activity of mutant p21 ras is, therefore, considered as the consequence of p21 ras being constitutively activated in its GTP-bound state. Thus, loss of function of GAPs to negatively regulate the activity of p21 ras might be important in the tumorigenesis process. Supporting this idea, mutations of the NF1 gene and reduced neurofibromin expression and catalytic activity were described in NF1-associated tumours and sporadic tumours of various types (Li et al, 1992; von Deimling et al, 1995). Decreased expression of p120 GAP or its shorter placental isoform has been demonstrated in benign and malignant human trophoblastic tumours (Stahle-Backdhal et al, 1995).

Meningiomas are tumours derived from leptomeningeal cells, specifically the arachnoid cap cells surrounding the brain and spinal cord. These tumours account for up to 20% of all primary intracranial neoplasms and 25% of intraspinal tumours (Russel and Rubinstein, 1989). Although meningiomas are usually benign, they often recur after seemingly complete surgical removal and occasionally progress to a fully malignant phenotype (Kujas, 1993). Clinically and histologically, meningiomas are a diverse group of tumours classified into different histological subtypes

*Received 29 October 1996*
*Revised 12 February 1997*
*Accepted 20 February 1997*

*Correspondence to:* M Golubic, Cleveland Clinic Foundation, Department of Neurosurgery, 9500 Euclid Avenue/NC2-150, Cleveland, OH 44195, USA

**Table 1** Clinical data and neurofibromin expression in sporadic meningiomas

| Tumour | Age (years) | Sex | Location | Histology | IB | IP/IB | IP/GTPase assay Ratio NF1/GAP | Maltoside sensitivity |
|---|---|---|---|---|---|---|---|---|
| 1 | 25 | F | Frontal fossa | Transitional (NF2) | *R* | *R* | *0.18* (*16.4*/88.9) | *R* |
| 2 | 77 | F | Spine | Meningotheliomatous | *R* | *R* | ND | + |
| 3 | 42 | F | Sphenoid wing | Meningotheliomatous | + | ND | ND | + |
| 4 | 36 | F | Posterior fossa | Transitional | + | ND | ND | + |
| 5 | 35 | F | Petroclival | Meningotheliomatous | + | ND | ND | + |
| 6 | 77 | F | Sphenoid wing | Meningotheliomatous | *R* | ND | ND | *R* |
| 7 | 48 | F | Sphenoid wing/infratemporal | Transitional | *R* | *R* | *0.59* (*42.4*/71.7) | *R* |
| 8 | 68 | F | Foramen magnum | Meningotheliomatous | *R* | *R* | *0.36* (*18.7*/52.1) | *R* |
| 9 | 70 | F | Frontoparietal convexity | Fibroblastic/psammoma bodies | + | + | 1.27 (97.1/76.5) | + |
| 10 | 69 | M | Tuberculum sella | Transitional | + | + | ND | ND |
| 11 | 46 | F | Sphenoid wing | Meningotheliomatous | + | + | 1.2 (79.5/66.2) | + |
| 12 | 41 | M | Frontoparietal convexity | Malignant | + | + | 1.26 (79.0/62.8) | + |
| 13 | 61 | F | Tentorial | Meningotheliomatous/secretory | + | + | 0.93 (81.2/86.8) | + |
| 14 | 74 | F | Parasagittal | Malignant | *R* | *R* | *0.56 (26.8/48.0)* | *R* |
| 15 | 68 | F | Spinal | Meningotheliomatous/psammoma bodies | + | + | 0.99 (75.2/75.3) | + |
| 16 | 67 | F | Occipital convexity | Transitional/infiltrating | + | + | 1.23 (88.1/71.6) | + |
| 17 | 54 | F | Parietal/occipital convexity | Malignant | + | + | 1.19 (77.8/65.2) | + |
| 18 | 48 | F | Petrous | Meningotheliomatous | + | *R* | *0.63* (*37.8*/59.7) | + |
| HB | | | | | + | + | 1.00 (100.0/100.0) | + |

IB, immunoblotting of neurofibromin; +, intensity of neurofibromin 250-kDa band is similar to brain tissue and established leptomeningeal cells LTAg2B; R, intensity of neurofibromin 250-kDa band is reduced in comparison with brain tissue and established leptomeningeal cells LTAg2B; IP/IB, neurofibromin immunoprecipitation by anti-NF1 (C1) serum followed by immunoblotting; IP/GTPase assay, neurofibromin immunoprecipitation followed by p21 ras immunoprecipitation GTPase assay; NF1, neurofibromin; GAP, p120 GAP; ratio NF1/GAP, percentage of GTPase activity of NF1 compared with human brain divided by the percentage of GTPase activity of p120 GAP compared with human brain (shown in brackets); HB, human brain; ND, experiment not done. Maltoside sensitivity: +, sensitivity is similar to that of the brain tissue; R, reduced sensitivity. The results different from normal are italicized.

(Scheithauer, 1990). Little, however, is known about the molecular mechanisms responsible for the development and histopathological heterogeneity of these tumours (Collins, 1990).

The NF2 gene seems to be the major meningioma gene because of its frequent mutational inactivation in sporadic meningiomas (Lutchman and Rouleau, 1996). The levels of the NF2 protein, termed schwannomin (Rouleau et al, 1993) or merlin (Trofatter et al, 1993), are severely reduced in almost 60% of sporadic meningiomas, as demonstrated in our recent analysis (Lee et al, 1997). Besides the NF2 gene, several candidate meningioma genes were identified (Murphy et al, 1993; Peyrard et al, 1994; Lekanne Deprez et al, 1995).

The possibility that the NF1 gene plays a role in meningioma development has not been explored experimentally. Such plausibility, however, is supported by at least two observations. Firstly, some pathological features of NF1 and loss of control of cell growth are apparent in neural crest-derived tissues (Basu et al, 1992; DeClue et al, 1992), and meninges are thought to be partly of mesenchymal and partly of neural crest origin (O'Rahilly and Mueller, 1986). Secondly, the involvement of NF1 gene-bearing chromosome 17 in meningioma development is suggested by some cytogenetic studies (Yamada et al, 1980; Maltby et al, 1988).

Mutational analysis of the NF1 gene is complicated by its large size, numerous exons and the presence of pseudogenes (Li et al, 1995). At least 80% of the identified mutations potentially encode truncated proteins because of premature translation termination (Heim et al, 1994; von Deimling et al, 1995). As such, the analysis of neurofibromin by specific antibodies could reveal the consequences of most NF1 mutations either by the presence of truncated proteins or by reduced expression of the full-length neurofibromin. The biochemical analysis of GAP activity of tumour-derived neurofibromin could be used to determine effects of mutations that might occur in the GRD of the protein. The analysis of protein has an additional advantage. In contrast to the ubiquitous expression of the NF1 gene, neurofibromin is predominantly expressed in the adult brain (Daston et al, 1992; Golubic et al, 1992). Therefore, neurofibromin derived from non-tumorous cells present in the tumour, such as lymphocytes, macrophages, endothelial cells, etc., would have only minor significance in the interpretation of the results.

In this study, we determined first the expression level of neurofibromin in established human leptomeningeal cells, LTAg2B, using immunoblotting experiments with two specific antibodies. Then, to test the hypothesis that the loss of function of neurofibromin is involved in meningioma development, we determined the expression levels and the GAP activity of neurofibromin upon p21 ras in one NF2-related meningioma and in 17 primary sporadic meningiomas.

### PATIENTS AND METHODS

### Tissue samples and cell lines

Human brain and tumour tissue specimens were obtained form the operating room with the patient's consent and approval of the Institutional Review Board (Cleveland Clinic Foundation IRB no. 5400). The NF2 patient, a 25-year-old woman, participating in this study met the criteria agreed upon in a consensus conference at the National Institutes of Health (Mulvihill et al, 1990). She presented with bilateral vestibular schwannomas and multiple intracranial meningiomas. Normal brain specimen was obtained from an epilepsy patient with no apparent structural lesion and who was undergoing temporal lobectomy. Diagnosis of meningiomas and their histopathological classification was determined by neuropathologists.

 *© Cancer Research Campaign 1997*

**Figure 1** Immunoblotting of soluble protein fraction of human brain tissue (HB), established human leptomeningeal cells LTAg2B (LTAg2B), murine fibroblast cells NIH3T3 (NIH) and the human astrocytoma CRT cell line (CRT) by anti-NF1GRP(N) IgG (top) and anti-p120 GAP IgG (bottom), as described in the Patients and methods section. The protein amount in micrograms (μg) that was analysed is indicated above the upper panel. Molecular size standards in kDa are indicated on the right. On the left, NF1 indicates neurofibromin band, while GAP indicates p120 GAP-specific band

The leptomeningeal cell line LTAg2B was established from a primary culture of human leptomeningeal cells transfected with an SV40 T antigen construct (Murphy et al, 1991). The cells were grown in minimum essential medium with Earle's salts (Gibco BRL) supplemented with 10% fetal calf serum and antibiotic–antimycotic agents. Murine fibroblast cells NIH 3T3 were grown in Dulbecco's modified eagle medium (DMEM) (Gibco BRL) supplemented with 10% calf serum and 1% penicillin/streptomycin. Human astrocytoma (CRT) cells, isolated from a grade IV glioblastoma, were grown in DMEM with 10% fetal calf serum (Estes et al, 1990).

### Preparation of tissue extracts

Immediately upon surgical excision tumour or normal human brain tissue was transported on ice to the laboratory in buffer A (50 mM Tris-HCl, pH 7.5, 1 mM EDTA, 1 mM DTT and a cocktail of protease inhibitors). Tumour tissue was homogenized using a Polytron PT 3000 (Brinkmann) in buffer A. The homogenate was centrifuged at 100 000 **g** for 60 min, and the supernatant containing the cytosolic, soluble proteins (S100 fraction) and pellet was saved. The pellet was briefly homogenized, washed twice with buffer A and resuspended in buffer A containing 1% Triton X-100. After a 30-min incubation on ice, the sample was centrifuged at 100 000 **g** for 60 min at 4°C. Supernatant (1% Triton fraction) was saved and stored in aliquots along with S100 fraction in an –85°C freezer. The protein concentration of the tumour samples was determined using BCA protein assay reagent from Pierce, according to the protocol specified by the manufacturer, with a bovine serum albumin (BSA) standard.

LTAg2B, NIH3T3 and CRT cells were grown to confluence, washed three times in ice-cold phosphate-buffered saline (PBS) and scraped off the plates in buffer A. Cell lysates were prepared by sonication (3 × 10 s, 35% output) in a sonic dismembrator (Fisher, Model 300). All subsequent steps were performed as described above for brain and meningioma tissue samples.

### Immunoblotting of neurofibromin and p120 GAP

Total proteins from prepared fractions of the tumour lysates and positive control tissue were separated using standard 7% sodium dodecyl sulphate (SDS)–polyacrylamide gel electrophoresis (SDS/PAGE) on a Bio-Rad Protean II 16-cm cell. As a positive control, S100 fraction of rabbit and human brain was used, as the

**Figure 2** Immunoblotting of soluble fraction of 18 meningiomas. A 100-μg aliquot of soluble fraction of human brain (HB) and 18 meningiomas (nos. 1–18, see Table 1) was immunoblotted with (**A**) anti-NF1GRP(N) IgG, (**B**) anti-NF1GRP(D) IgG, (**C**) anti-p120 GAP IgG and (**D**) anti-actin IgG. Molecular size standards in kDa are indicated on the left. On the right, NF1(N) indicates neurofibromin recognized by anti-NF1GRP(N) IgG, NF1(C) indicates neurofibromin detected by anti-NF1GRP(D) IgG, and GAP stands for p120 GAP-specific band. Tumour samples nos. 13–18 are from a different gel, with longer exposure in the case of anti-NF1GRP(N) IgG and anti-actin immunoblotting

© Cancer Research Campaign 1997

**Figure 3** Detection of neurofibromin by immunoprecipitation followed by immunoblotting. (**A**) Neurofibromin (marked NF1 on the right) was immunoprecipitated from 4 mg of soluble fraction of rabbit brain (RB) and meningiomas (tumour number is indicated below) by anti-NF1GRP(N) IgG (marked as N above the gel) or anti-NF1GRP(D) IgG (marked as C above) and immunoblotted with anti-NF1GRP(N) IgG. The first lane in which non-immune serum was used for immunoprecipitation is indicated by non-imm. above. Immunoprecipitation reactions with only 1 mg of protein are indicated by an asterisk. (**B**) Immunoprecipitation analysis of NF2-related meningioma (tumour no. 1). In lanes 2, 4, 6 and 8, immunoprecipitation was performed with the non-immune serum. In lanes 1 and 3, immunoprecipitation was performed with anti-NF1(C1) serum and in lanes 5 and 7 with polyclonal anti-p120 GAP IgG. A total of 4 mg of NF2-related meningioma (lanes 1, 2, 5 and 6) and rabbit brain (lanes 3, 4, 7 and 8) as a positive control were used. Immunoblotting of immunoprecipitated proteins was performed with anti-NF1(C1) serum (lanes 1–4) and anti-p120 GAP IgG (lanes 5–8). (**C**) Immunoprecipitation analysis of meningioma no. 2 lysate. Immunoprecipitation was performed by non-immune serum (lane 1) and by anti-NF1(C1) serum from 1 mg of soluble protein from rabbit brain (lane 2) and meningioma no. 2 (lane 3). Immunoblotting was done with anti-NF1(C1) serum. NF1 indicates the neurofibromin-specific band. (**D**) Immunoprecipitation was performed by anti-NF1GRP(N) IgG (indicated by N above) and anti-NF1(C1) serum (indicated by C above) from 2 mg of protein of rabbit brain (RB). A total of 2 mg of protein from meningiomas, with tumour number indicated at the bottom of the figure, was immunoprecipitated with anti-NF1(C1) serum (top) or with anti-p120 GAP IgG (bottom). Neurofibromin and p120 GAP immunoprecipitates were first used to measure their GTPase stimulatory activity and then boiled in Laemmli buffer followed by SDS/PAGE. Immunoblotting was done using anti-NF1(C1) serum (top) and anti-p120 GAP (bottom)

non-tumorous tissue from the patients was not available. The protein samples were run along with prestained protein standards and transferred by a semi-dry transfer cell (Trans-blot SD, Bio-Rad) to supported nitrocellulose membrane (Schleicher & Schuell) in buffer containing 48 mM Tris, 39 mM glycine, 0.0375% SDS and 20% methanol, pH 9.2. The nitrocellulose filters were incubated in blocking solution (5% dry milk, 10 mM Tris-HCl, pH 8.0, 150 mM sodium chloride, 0.05% Tween 20) for 1 h at room temperature. Two commercially available polyclonal anti-neurofibromin antibodies (Santa Cruz Biotechnology) raised in rabbits against two peptides were diluted in blocking buffer to the final concentration of 0.25 μg $ml^{-1}$. The first anti-neurofibromin antibody designated anti-NFlGRP(N) IgG recognizes an epitope localized at the amino terminus of the human neurofibromin (amino acid residues 509–528), while anti-NFlGRP(D) antibody is specific for the epitope at the carboxy terminus (residues 2798–2818) of human

 © Cancer Research Campaign 1997

**Figure 4** Analysis of total (grey bars) and maltoside-inhibited GAP activity (solid bars) of soluble fraction of meningiomas. (**A**) Ras '0' indicates percentage of GTP that remains associated with p21 ras at zero time, before incubation. Ras indicates percentage of GTP after an incubation of p21 ras for 15 min at 30°C, as described in the Patients and methods section. Incubation in the presence of 1.25 mM dodecyl maltoside (neurofibromin-specific inhibitor) does not affect the intrinsic GTPase activity of p21 ras. The addition of 250 µg of soluble fraction of rabbit brain (RB) and meningiomas nos. 13, 15 and 18 dramatically stimulates intrinsic GTPase activity of p21 ras, which is strongly inhibited by incubation in the presence of maltoside. The total and maltoside-inhibited GAP activity of meningiomas no. 7 and no. 8 is reduced, indicating diminished GAP activity of neurofibromin. Results shown are averages of two determinations, with standard deviations less than 5%. The results shown in (**B**) are from the dose–response experiment in which 100–1000 µg of soluble protein from menigiomas were assayed. Only the results for the 500-µg data point are shown, except for meningioma no. 6 (1080 µg). In meningiomas nos. 1, 6 and 14, GAP activity was poorly inhibited by maltoside, indicating reduced GAP activity of neurofibromin in these tumours neurofibromin. Incubation with the primary antibodies was done overnight at 4°C. The blots were washed twice in Tris-buffered saline (TBS) (10 mM Tris-HCl pH 8.0, 150 mM sodium chloride) with 0.05% Tween 20 for 7 min. The secondary anti-rabbit-horseradish peroxidase conjugated antibody (Boehringer) was diluted 1:2000 in blocking buffer and incubated with the blots for 1 h at room temperature. The membranes were washed three times for 5 min with TBS, 0.05% Tween 20 and washed once for 5 min with TBS. The proteins were detected using Western blot chemiluminiscence reagents (Renaissance, DuPont NEN).

Stripping of the anti-neurofibromin antibodies from membranes was performed for 30 min at 50°C in stripping buffer (62.5 mM Tris-HCl pH 6.8, 2% SDS, 100 mM 2-mercaptoethanol). The membranes were then washed four times for 5 min in TBS, 0.05% Tween 20 and incubated for 1 h at room temperature in blocking buffer (1% BSA in 10 mM Tris-HCl, pH 7.5, 100 mM sodium chloride, 0.1% Tween 20).

Mouse monoclonal anti-p120 GAP antibody was obtained from Zymed and used according to the provided instructions. The secondary anti-mouse-horseradish peroxidase conjugated antibody (Boehringer) was diluted 1:5000 in 5% milk blocking buffer and incubated with the blots for 1 h at room temperature. Subsequent steps were performed as described for anti-neurofibromin immunoblotting.

Mouse monoclonal antibody to actin (Boehringer) was diluted in the blocking buffer (final concentration 0.5 µg $ml^{-1}$) and incubated at 4°C overnight. Washing and subsequent steps were done as described for neurofibromin and p120 GAP immunoblotting.

### Immunoprecipitation of neurofibromin and p120 GAP

To immunoprecipitate neurofibromin from tumour and control extracts, sodium chloride was added to obtain a final concentration of 100 mM along with the corresponding antibody. Anti-neurofibromin anti-NF1GRP(N) IgG and anti-NF1GRP(D) IgG were used at 1 µg per sample. The most efficient immunoprecipitation of neurofibromin, however, was achieved with anti-NF1(C1) polyclonal serum specific for the epitope at the carboxy terminus (residues 2798–2814) of human neurofibromin. This anti-neurofibromin serum was raised in our laboratory and was successfully used in immunoblotting and immunoprecipitation experiments using soluble and particulate fraction extracts of several murine organs (Golubic et al, 1992). In the present study, 10 µl of serum was used per immunoprecipitation reaction. Two negative controls were used in the immunoprecipitation experiments. The first was immunoprecipitation with 10 µl of non-immune rabbit serum. In the second case anti-neurofibromin antibodies were neutralized by preincubation with 10-fold (by weight) excess of specific peptide antigen in PBS for 2 h at room temperature. For p120 GAP immunoprecipitation, a commercial anti-human p120 GAP polyclonal antibody was used (Upstate Biotechnology). This antibody was raised against GAP fusion protein containing amino acids 171–448 of human GAP and was used at 10 µg per sample. The incubation with antibodies was done at 4°C overnight. About 35 µl of protein A Sepharose slurry (50% in buffer A) was added, and the samples were rotated on the wheel for 40 min at 4°C. The beads with immunoprecipitated neurofibromin were washed twice in buffer A containing 0.2% Nonidet-P40 and 100 mM sodium chloride, twice in buffer A containing 100 mM sodium chloride and once in buffer A. The p120 GAP immunoprecipitates were washed twice in buffer A containing 0.1% Nonidet-P40 and 75 mM sodium chloride, twice in buffer A containing 75 mM sodium chloride and once in buffer A. To reduce the background, the beads were settled down by gravity, except for the last wash when Eppendorf minifuge was used. The beads were then boiled in 1 × Laemmli buffer for 2 min and proteins were separated on SDS/PAGE. Protein transfer and immunoblotting were performed as described above.

### GTPase assay with meningioma protein extracts

The p21 ras GTPase stimulatory activity of the S100 fraction of meningiomas was determined by p21 ras immunoprecipitation assay.

© Cancer Research Campaign 1997

**Figure 5** Immunoprecipitation of neurofibromin (grey bars) and p120 GAP (black bars) from the soluble fraction of meningiomas followed by GTPase assay. Ras indicates p21 ras 'zero' time (see Figure 4 legend), while N-im, indicates immunoprecipitation with non-immune serum from human brain tissue (HB). The numbers indicate each meningioma sample used for immunoprecipitation (see Table 1)

Briefly, purified bacterially synthesized c-Ha-Ras was incubated for 5 min at 30°C with 1 μM [α-$^{32}$P]GTP (3000 Ci mmol$^{-1}$; DuPont NEN) in buffer containing 20 mM Tris-HCl, pH 7.5, and 1 mM DTT. GTPase reaction was initiated by addition of magnesium chloride and the meningioma lysate in 60 μl of reaction buffer (final concentrations: 20 mM Tris-HCl, pH 7.5, 1mM DTT, 5 mM magnesium chloride, 0.5 mM unlabelled GTP). The final concentration of p21 ras in the reaction was 30 nM. After incubation at 30°C for 15 min, p21 ras was immunoprecipitated by rat monoclonal antibody Y13-259 and protein A Sepharose beads coated with rabbit antibody to rat IgG. Bound nucleotides were released from the immunoprecipitates by boiling for 2 min in a buffer containing 1 mM EDTA and 0.35% SDS. The nucleotides were resolved on a polyethyleneimine cellulose thin-layer chromatography plate with a 1 M potassium phosphate, pH 3.4, mobile phase. The separated nucleotides were visualized and their intensity was determined using phosphoimager analysis.

### GTPase assay with immunoprecipitated neurofibromin and p120 GAP

The p21 ras GTPase stimulatory activity of immunoprecipitated neurofibromin or p120 GAP was determined by p21 ras immunoprecipitation assay (Golubic et al, 1992). Briefly, protein A–Sepharose beads with immunoprecipitated neurofibromin or p120 GAP were incubated for 15 min at 30°C in the reaction buffer containing p21 ras, as described above. After incubation, rat monoclonal antibody Y13-259 and protein A–Sepharose beads coated with rabbit antibody to rat IgG were added to the supernatant. All subsequent steps were as described above.

### RESULTS

### Neurofibromin expression in established human leptomeningeal cell line LTAg2B

The level of neurofibromin expression was first determined in established human leptomeningeal cells LTAg2B, from which meningiomas are thought to develop (Russel and Rubinstein, 1989). We have shown earlier that neurofibromin is predominantly expressed and catalytically active in the soluble fraction of the rat brain (Golubic et al, 1992). Therefore, the soluble fractions of LTAg2B cells, human brain tissue, murine NIH3T3 fibroblast cells and human astrocytoma CRT cells were immunoblotted by anti-NF1GRP(N) IgG, which recognizes an epitope localized at the amino terminus of the human neurofibromin (Figure 1, top). As expected, a neurofibromin-specific band of about 250 kDa was detected in human brain tissue. The expression level of neurofibromin in the soluble fraction of leptomeningeal cells LTAg2B was reduced about twofold in comparison to the brain sample, as determined by densitometry (not shown). It was however more than 10-fold higher than in NIH 3T3 fibroblast and astrocytoma CRT cells. Immunoblotting of the same membrane with an anti-p120 GAP antibody (after stripping of anti-neurofibromin antibody) revealed that the expression level of p120 GAP differed little among the samples analysed (Figure 1, bottom).

### Neurofibromin expression in meningiomas – immunoblotting experiments

Neurofibromin expression levels in meningiomas were determined by immunoblotting of the soluble fraction of the tumours. The clinical and histopathological characteristics of 17 sporadic meningiomas and one NF2-related tumour analysed in this study are summarized in Table 1. In immunoblotting experiments with anti-NF1GRP(N) IgG, neurofibromin was detected in the human brain tissue and in most meningiomas (Figure 2A). In some tumours (meningiomas nos. 1, 2, 6, 7, 8 and 14), however the specific 250-kDa band intensity was reduced compared with human brain tissue and other meningiomas (Figure 2A). Although immunoblotting with anti-NF1GRP(N) IgG could, theoretically, detect truncated neurofibromin molecules larger than 70 kDa, no specific band of size smaller than the 250-kDa band of full-length neurofibromin was seen in the tumours analysed (not shown).

Neurofibromin was also detected as a 250-kDa band in human brain tissue and most meningiomas by anti-NF1GRP(D) IgG (Figure 2B). In this case, the specific band was barely detected in tumours nos. 1, 2, 6, 7 and 8, while the intensity of the neurofibromin band in other meningiomas was similar to those of human brain and other meningiomas. In addition to the 250-kDa band, a faster migrating band of about 200 kDa was detected in meningiomas but not in human brain tissue (Figure 2B). The identity of that band and its relationship to neurofibromin is not known. Immunoblotting experiments in which anti-NF1GRP(D) IgG was preincubated with homologous peptide suggest that this band is non-specific (not shown).

The expression of p120 GAP in the soluble fraction of meningiomas was analysed using a commercial monoclonal antibody (Figure 2C). In contrast to the reduced neurofibromin expression seen in six meningiomas, all tumours expressed p120 GAP at levels similar or slightly reduced compared with that observed in human brain tissue. The only exception was tumour no. 14, in which the intensity of the 120-kDa-specific band was severely reduced compared with human brain tissue and other meningiomas. Immunoblot analysis of tumour samples using anti-actin antibody demonstrates that equal amounts of protein samples were used (Figure 2D). Detection of the single p120 GAP and actin-specific band also suggests that non-specific protein degradation of meningioma lysates is not the cause of the reduced neurofibromin levels

© Cancer Research Campaign 1997

found in the six meningiomas. The staining of the soluble proteins from tumours with reduced neurofibromin using Coomassie blue also showed intact high-molecular-weight proteins and no signs of protein degradation (not shown). The summary of immunoblotting results is shown in Table 1. Similar results were obtained when neurofibromin, expressed in the 1% Triton X-100 fraction of the meningiomas, was analysed by immunoblotting (not shown).

### Neurofibromin expression in meningiomas – immunoprecipitation followed by immunoblotting experiments

In addition to immunoblotting, the expression level of neurofibromin in meningiomas was determined by immunoprecipitation of neurofibromin from the soluble fraction of the tumours followed by immunoblotting with anti-neurofibromin antibodies. As the availability of human brain tissue was limited, for immunoprecipitation experiments a soluble fraction of rabbit brain was used as a positive control. Figure 3A shows the results obtained after immunoprecipitation of neurofibromin from 4 mg of soluble fraction of several meningiomas with anti-NF1GRP(N) IgG or anti-NF1GRP(D) IgG followed by immunoblotting with anti-NF1GRP(N) IgG. Immunoblotting of immunoprecipitated neurofibromin from seven meningiomas revealed a strong 250-kDa-specific band in five tumours (nos. 9, 10, 11, 12 and 17) (Figure 3A). In contrast to immunoblotting, anti-NF1GRP(D) IgG was much more efficient in immunoprecipitation than anti-GRP(N) IgG. Little neurofibromin, however, was immunoprecipitated even with anti-NF1GRP(D) IgG from meningiomas no. 7 and no. 8, thus confirming the observation made by immunoblotting (see Figure 2A and B). Very little, if any, neurofibromin was immunoprecipitated from meningioma no. 1 (Figure 3B), no. 2 (Figure 3C) and tumours no. 14 and no. 18 (Figure 3D) with anti-neurofibromin anti-NF1(C1) serum specific for the carboxy terminus of the protein.

In contrast to the reduced level of neurofibromin seen in meningiomas nos. 1, 2, 7, 8, 14 and 18 by immunoprecipitation, the intensity of immunoprecipitated p120 GAP was similar in all tumours analysed (see Figure 3B and D for tumours nos. 1, 7 and 18; for other tumours data are not shown). The only exception was meningioma no. 14, in which p120 GAP was significantly reduced (Figure 3D).

In addition to neurofibromin, in some meningiomas (see tumours no. 7 and no.11 in Figure 3A; no. 13 and no. 18 in Figure 3D) a faster migrating band of about 200 kDa was also immunoprecipitated with both antibodies. The relationship of that protein to neurofibromin, however, is unknown. The presence of this band did not correlate with the reduction of neurofibromin, as it is detected in meningiomas no. 11 and no. 13, which expressed normal levels of neurofibromin. No other specific band of smaller size was detected by immunoprecipitation in any meningioma.

### GTPase-activating protein (GAP) activity of neurofibromin from meningioma lysates

At present, stimulation of p21 ras GTPase activity is the only known function of neurofibromin (Xu et al, 1990; Golubic et al, 1992), and it was therefore used as another measure of neurofibromin levels in meningiomas. The GAP activity of neurofibromin from the soluble fraction of meningiomas was determined by p21 ras GTPase assay. A detergent dodecyl maltoside potently inhibits GAP activity of neurofibromin but not that of p120 GAP (Bollag and McCormick, 1991) and was used at a concentration of 1.25 mM to determine the contribution of neurofibromin to the total GAP activity of meningioma lysates. The total GAP activity minus maltoside-insensitive activity is due to neurofibromin. The total and dodecyl maltoside-susceptible GAP activity of the soluble fraction of 18 meningiomas (tumours no. 1 through to no. 18) and rabbit brain as a positive control were determined (Figure 4A and B).

The GTPase stimulatory activity of tumours nos. 2, 5, 9, 11, 13, 15 and 18 contained maltoside-susceptible GAP activity similar to that seen in brain tissue (Figure 4A and B). In five meningiomas (nos. 1, 6, 7, 8 and 14), GAP activity was only weakly susceptible to inhibition by maltoside (Figure 4A and B) and therefore contained little GTPase-stimulatory activity upon p21 ras that could be attributed to neurofibromin.

Although results of this functional analysis of neurofibromin mostly correlated well with the results of the previous experiments (exceptions were tumours no. 2 and no. 18), their interpretation is complicated by recent identification of other p21 ras-specific GAP proteins in the brain tissue (Maekawa et al, 1994; Weisbach et al, 1994; Baba et al, 1995). At present, it is not known whether these proteins are expressed in meningiomas and whether their GAP activity is susceptible to inhibition by maltoside.

### GAP activity of immunoprecipitated neurofibromin

To eliminate the possibility that other p21 ras-specific GAP molecules (Maekawa et al, 1994; Weisbach et al, 1994; Baba et al, 1995), besides neurofibromin and p120 GAP, contribute to the total and maltoside-sensitive GAP activity of meningioma lysates, neurofibromin and p120 GAP were immunoprecipitated from the soluble fraction of meningiomas, and the GAP activity of immunoprecipitated proteins was determined in p21 ras GTPase assay.

Neurofibromin was immunoprecipitated from 4 mg of the soluble fraction from 12 meningiomas with the anti-NF1(C1) polyclonal antibody specific for the epitope at the carboxy terminus of human neurofibromin. The GAP activity of immunoprecipitated neurofibromin was high in meningiomas that had previously shown good maltoside-susceptible GAP activity (tumours nos. 9–13 and 15–17). The average GAP activity of immunoprecipitated neurofibromin in these meningiomas was 83% (range 75–97%; standard deviation 7.5) of the activity detected in neurofibromin immunoprecipitated from the soluble fraction of human brain (Figure 5 and Table 1). In contrast, GAP activity of immunoprecipitated neurofibromin from meningiomas nos. 1, 7, 8, 14 and 18 was substantially reduced (Figure 5 and Table 1). Immunoprecipitated neurofibromin from meningiomas nos. 1, 8 and 14 had only 16%, 19% and 27% of GAP activity of neurofibromin from human brain tissue respectively (Table 1). The reduction of neurofibromin's GAP activity from tumours no. 7 and no. 18 was less severe (42% and 38% respectively). Results similar to those in Figure 5 and Table 1 were obtained when GAP activity of neurofibromin was determined after its immunoprecipitation from 2 mg of soluble protein from meningiomas (not shown).

Immunoprecipitation with anti-p120 GAP antibody was also performed as a control. In contrast to the substantial reduction of GAP activity of immunoprecipitated neurofibromin from meningiomas nos. 1, 7, 8, 14 and 18, the p120 GAP GTPase stimulatory activity was either unchanged or slightly reduced in these tumours compared with other meningiomas with good neurofibromin expression (see Figure 5 and Table 1). To better estimate the degree of neurofibromin reduction in these meningiomas, the ratio between the GAP activity of neurofibromin and p120 GAP was calculated (see Table 1). Compared with the neurofibromin–p120 GAP ratio defined as 1.00 in human brain tissue, three tumours (nos. 7, 14 and 18) had a ratio of about 0.6. Tumours no. 1 and no. 8 had an even smaller neurofibromin–p120 GAP ratio of 0.18 and 0.36 respectively. All other meningiomas tested had an average ratio of 1.15 (range 0.99–1.27). These experiments conclusively confirmed the conspicuous reduction of neurofibromin and its GAP activity in meningiomas nos. 1, 7, 8 and 14 observed throughout this study.

### DISCUSSION

This study resulted in several novel observations concerning the role that neurofibromin might play in meningioma tumorigenesis. After extensive analysis using four different methods, we show for the first time that neurofibromin is expressed at high levels in leptomeningeal cells and in sporadic meningiomas, their tumour derivatives. Furthermore, reduced expression and consequently diminished GAP activity of neurofibromin were found in about 28% of the tumours analysed.

Neurofibromin expression in the adult nervous system was found by immunostaining of tissue sections limited to neurons, oligodendrocytes and non-myelinating Schwann cells (Daston et al, 1992). This study provides evidence that neurofibromin is also highly expressed in the established human leptomeningeal cell line. Although LTAg2B cells might differ from native leptomeningeal cells, at least by morphology and expression of tissue markers, they are indistinguishable from arachnoid cells of leptomeninges (Murphy et al, 1991). As determined by immunoblotting, these cells abundantly expressed full-length neurofibromin at levels 10-fold higher than murine NIH 3T3 fibroblast and human astrocytoma cell line CRT. In contrast, the expression level of p120 GAP was found to be similar among these cells. The high expression level of neurofibromin in the leptomeningeal cell line suggests that functions of neurofibromin in these cells could be physiologically important, as already shown for Schwann cells (Basu et al, 1992; DeClue et al, 1992; Takahashi et al, 1995) and neurons (Vogel et al, 1995).

Based on results obtained by immunoprecipitation and immunoblotting experiments, we concluded that 11 out of 18 meningiomas expressed neurofibromin at levels similar or slightly reduced compared with human brain tissue. The results of the GTPase assay with the soluble fraction of meninigiomas and neurofibromin immunoprecipitated from these tumours were compatible with the neurofibromin expression data (Table 1).

In five tumours (meningioma nos. 1, 6, 7, 8 and 14), the expression levels of neurofibromin and neurofibromin's GAP activity were reduced in comparison to other meningiomas. Reduced expression of neurofibromin was also found in meningioma no. 2 by immunoblotting with amino and carboxy terminus-specific antibodies (Figure 2) and immunoprecipitation with the antibody recognizing an epitope at the carboxy terminus of the molecule (Figure 3C). In tumour no. 18, reduced neurofibromin was detected only by immunoprecipitation with antibody directed against the carboxy terminus of the protein (Figure 3D and Table 1). Consequently, GAP activity of immunoprecipitated neurofibromin from tumour no. 18 was reduced (meningioma no. 2 was not analysed using this assay) (Figure 5 and Table 1). Examination of the maltoside-susceptible GAP activity of these two meningiomas, however, suggested that neurofibromin is catalytically active at levels similar to other meningiomas with normal neurofibromin expression (Figure 4). The discrepancy in tumour no. 18 could be explained by the presence of an altered epitope site at the carboxy terminus of neurofibromin that prevented efficient immunoprecipitation. The conflicting finding of the obvious reduction of neurofibromin expression and the presence of maltoside-susceptible activity in meningioma no. 2 can be explained by the presence of maltoside-susceptible GAP activity that is distinct from neurofibromin. It is not known, however, whether other p21 ras-specific GAPs are susceptible to inhibition by maltoside (Maekawa et al, 1994; Weisbach et al, 1994).

In contrast to alterations found in neurofibromin, the expression levels and catalytic activity of the other p21 ras-specific GTPase stimulatory protein, p120 GAP, were similar in all meningiomas except in malignant tumour no. 14. In this tumour, both neurofibromin and p120 GAP were reduced. The reduction of GAP activity of neurofibromin, however, was more severe than that of p120 GAP (Table 1).

No association between the age of meningioma detection and neurofibromin status was found. There was also no clear correlation between tumour location and reduced expression of neurofibromin. Reduced neurofibromin expression and its GAP activity were found in all histological types of sporadic tumours examined, except in a single fibroblastic meningioma. This occurred in meningiomas of meningotheliomatous (four out of nine, 44%), transitional (two out of five, 40%) and malignant (one out of three, 33%) histological types. The reduced neurofibromin expression and GAP function was therefore not associated with any particular histological type. Surprisingly, a meningioma derived from an NF2 patient had a severely reduced expression level and GAP activity of neurofibromin. As expected, the analysis of protein extracts from that tumour indicated an absence of schwannomin/merlin (not shown).

Recently we found that frequency of schwannomin/merlin reduction in meningotheliomatous meningiomas was significantly lower than in other histological tumour types (Lee et al, 1997). This suggests that development of meningotheliomatous meningiomas is probably linked with alterations in other oncogenes or tumour-suppressor genes. Interestingly, three of four meningotheliomatous tumours with altered neurofibromin (nos. 6, 8 and 18) expressed normal levels of schwannomin/merlin (Lee et al, 1997). One interesting possibility is that neurofibromin and schwannomin/merlin control similar or interacting biochemical intracellular pathways and that disruption of either one contributes to meningioma development.

Both neurofibromin and schwannomin/merlin might be involved in signalling through the p21 ras pathway. Recent study suggests that the tumour-suppressor activity of schwannomin/merlin could be mediated through its anti-p21 ras function (Tikoo et al, 1994). Neurofibromin is considered to act as a negative regulator of p21 ras (Lowy and Willumsen, 1993) and as its effector (Moodie et al, 1995). Reduced neurofibromin expression in meningiomas could lead to changes in both functions because five meningiomas with reduced catalytic activity of neurofibromin expressed little of the full-length protein. No meningioma with a normal neurofibromin expression level and impaired GAP activity was found, suggesting that GRD of neurofibromin in these tumours was functionally intact. Therefore, it is likely that the inactivating mutations described in GRD of neurofibromin in other tumour types (Li et al, 1992) are absent or are rare in meningiomas.

The lack of knowledge about p21 ras expression and alterations in meningiomas further obscures the role of neurofibromin in p21

© Cancer Research Campaign 1997

ras signalling in meningiomas (Salgaller et al, 1990; Arvanitis et al, 1991). More data indirectly suggest p21 ras importance. The strong inappropriate expression (compared with normal leptomeningeal cells) of polypeptide growth factors and their receptors on the same population of meningioma cells suggests the important possibility of autocrine or paracrine functions for these factors (Todo et al, 1996). Because extracellular growth factors found in meningiomas act through protein tyrosine kinase receptors and activate p21 ras, the reduced expression of neurofibromin and the consequent decrease in both the negative regulation of p21 ras and/or its effector activity might be of importance in the tumorigenesis of meningiomas. If neurofibromin is the major regulator of p21 ras in leptomeningeal cells or meningiomas, as in the Schwann cells (Basu et al, 1992; DeClue et al, 1992), then the diminished GTPase stimulatory activity of neurofibromin would result in an increase of the proportion of p21 ras bound to GTP. Neurofibromin, however, does not stimulate GTPase activity of oncogenically activated p21 ras (Trahey and McCormick, 1987). It remains to be demonstrated whether oncogenic mutations in p21 ras occur in meningiomas and whether proliferation of meningiomas is dependent on the presence of the functional p21 ras.

### ACKNOWLEDGEMENTS

We thank Donna L George for generously providing established human leptomeningeal cells LTAg2B. We would also like to thank Dorthy Herzberg for editorial assistance in preparation of this manuscript and Jim Lang for excellent photographic work. We wish to thank Dr Masahiro Hitomi, Dr Guan Chen and Dr Alan Wolfman for critically reviewing the manuscript and for their constructive comments. This work has been supported by the National Institutes of Health (GM 52271) awarded to DWS, by an American Institute for Cancer Research grant (no. 94B63) awarded to MG and by the Department of Neurosurgery, The Cleveland Clinic Foundation, Cleveland, OH, USA.

### REFERENCES

Arvanitis D, Malliri AD, Antoniou D, Linardopoulos S, Field JK and Spandidos DA (1991) Ras p21 expression in brain tumors: Elevated expression in malignant astrocytomas and glioblastoma multiforme. *In vivo* **5**: 317–322

Baba H, Fuss B, Urano J, Poullet P, Watson JB, Tamanoi F and Macklin WB (1995) GapIII, a new brain-enriched member of the GTPase-activating protein family. *J Neurosci Res* **41**: 846–858

Ballester R, Marchuk D, Boguski M, Saulino A, Letche R, Wigler M and Collins F (1990) The NF1 locus encodes a protein functionally related to mammalian GAP and yeast IRA proteins. *Cell* **63**: 851–859

Basu TN, Gutmann DH, Fletcher JA, Glover TW, Collins FS and Downward J (1992) Aberrant regulation of ras proteins in tumor cells from type 1 neurofibromatosis patients. *Nature* **356**: 713

Bernards A, Haase VH, Murthy AE, Menon A, Hannigan GE and Gusella FJ (1992) Complete human NF1 cDNA sequence: two alternatively spliced mRNAs and absence of expression in a neuroblastoma line. *DNA Cell Biol* **11**: 727–734

Bollag G and McCormick F (1991) Differential regulation of rasGAP and neurofibromatosis gene product activities. *Nature* **351**: 576–579

Boss JL (1989) Ras oncogenes in human cancer: a review. *Cancer Res* **49**: 4682–4689

Collins VP, Nordenskjold M and Dumanski JP (1990) The molecular genetics of meningiomas. *Brain Pathol* **1**: 19–24

Daston MM, Scrable H, Norlund M, Sturbaum AK, Nissen LM and Ratner N (1992) The protein product of the neurofibromatosis type 1 gene is expressed at highest abundance in neurons, Schwann cells and oligodendrocytes. *Neuron* **8**: 415–428

Declue JE, Papagaeorge AG, Fletcher JA, Diehl SR, Ratner N, Vass WC and Lowy DR (1992) Abnormal regulation of mammalian p21 ras contributes to malignant tumor growth in von Recklinghausen (type 1) neurofibromatosis. *Cell* **69**: 265–273

Estes ML, Ransohoff RM, McMahon JT, Jacobs BS and Barna BP (1990) Characterization of adult human astrocytes derived from explant culture. *J Neurosci Res* **27**: 697–705

Golubic M, Roudebush M, Dobrowolski S, Wolfman A and Stacey DW (1992) Catalytic properties, tissue and intracellular distribution of neurofibromin. *Oncogene* **7**: 2151–2159

Heim RA, Silverman LM, Farber RA, Kam-Morgan LNW and Luce M (1994) Screening for truncated NF1 proteins. *Nature Genet* **8**: 218–219

Kujas M (1993) Meningioma. *Current Opin Neurol Neurosurg* **6**: 882–887

Lee JH, Sundaram V, Stein DJ, Kinney SE, Stacey D and Golubic M (1997) Reduced expression of schwannomin/merlin in human sporadic meningiomas. *Neurosurgery* **40**: 578–587

Lekanne Deprez RH, Riegman PHJ, Groen NA, Warringa UL, van Biezen NA, Molijn AC, Bootsma D, De Jong PJ, Menon AG, Kley NA, Seizinger BR and Zwarthoff EC (1995) Cloning and characterization of MN1, a gene from chromosome 22q11, which is disrupted by a balanced translocation in a meninigioma. *Oncogene* **10**: 1521–1528

Li Y, Bollag G, Clark R, Stevens J, Conroy L, Fullts D, Ward K, Friedman E, Samowitz W, Robertson M, Bradley P, McCormick F, White R and Cawthon R (1992) Somatic mutations in the neurofibromatosis 1 gene in human tumors. *Cell* **69**: 275–281

Li Y, O'Connell P, Breidenbach HH, Cawthon R, Stevens J, Xu G, Neil S, Robertson M, White R and Viskochil D (1995) Genomic organization of the neurofibromatosis type 1 gene (NF1). *Genomics* **25**: 9–18

Lowy DR and Willumsen BM (1993) Function and regulation of ras. *Annu Rev Biochem* **62**: 851–891

Lutchman M and Rouleau GA (1996) Neurofibromatosis type 2: a new mechanism of tumor suppression. *Trends Neurosci* **19**: 373–377

Maekawa M, Li S, Iwamatsu A, Morishita T, Yokota K, Imai Y, Koshaka S, Nakamura S and Hattori S (1994) A novel mammalian Ras GTPase-activating protein which has phospholipid-binding and Btk homology regions. *Mol Cell Biol* **14**: 6879–6885

Maltby EL, Ironside JW and Battersby RDE (1988) Cytogenetic studies in 50 meningiomas. *Cancer Genet Cytogenet* **31**: 199–210

Marchuk DA, Saulino AM, Tavakkol R, Swaroop M, Wallace MR, Andersen LB, Mitchekk AL, Gutmann DH, Boguski M and Collins FS (1991) cDNA cloning of the type 1 neurofibromatosis gene: complete sequence of the NF1 gene product. *Genomics* **11**: 931–940

Martuza RL and Eldridge R (1988) Medical progress: neurofibromatosis 2 (bilateral acoustic neurofibromatosis). *New Engl J Med* **318**: 684–688

Moodie SA, Paris M, Villafranca E, Kirshmeier P, Willumsen BM and Wolfman A (1995) Different structural requirements with the switch II region of the Ras protein for interactions with specific downstream targets. *Oncogene* **11**: 447–454

Mulvihill JJ, Parry DM, Sherman JL, Pikus A, Kaiser-Kupfer MI and Eldridge R (1990) Neurofibromatosis 1 (Recklinghausen disease) and neurofibromatosis 2 (bilateral acoustic neurofibromatosis). *Ann Int Med* **113**: 39–52

Murphy M, Chen J-N and George DL (1991) Establishment and characterization of a human leptomeningeal cell line. *J Neurosci Res* **30**: 475–483

Murphy M, Pykett MJ, Harnish P, Zang KD and George DL (1993) Identification and characterization of genes differentially expressed in meningiomas. *Cell Growth Diff* **4**: 725–722

O'Rahilly R and Mueller F (1986) The meninges in human development. *J Neuropathol Exp Neurol* **45**: 588–608

Peyrard M, Fransson I, Xie Y-G, Han F-Y, Ruttledge MH, Swahn S, Collins JE, Dunham I, Collins VP and Dumanski JP (1994) Characterization of a new member of the human β-adaptin gene family from chromosome 22q12, a candidate meningioma gene. *Hum Mol Genet* **3**: 1393–1399

Riccardi VM (1992) *Neurofibromatosis: Phenotype, Natural History and Pathogenesis*, 2nd edn. pp. 63–85. The Johns Hopkins University Press; Baltimore

Rouleau GA, Merel P, Lutchman H, Sanson M, Zucman J, Marineau C, Hoang-Xuan K, Demczuk S, Desmaze C, Plougaste B, Pulst SM, Lenoir G, Bijlsma E, Fashold R, Dumanski J, De Jong P, Parry D, Eldrige R, Aurias A, Delattre O and Thomas G (1993) Alteration in a new gene encoding a putative membrane-organizing protein causes neurofibromatosis type 2. *Nature* **363**: 515–520

Russel DS and Rubinstein LJ (1989) Tumours of the meninges and related tissues. In *Pathology of Tumours of the Nervous System*, Russel DS and Rubinstein LJ. (eds), pp. 449–532. Williams & Wilkins: Baltimore

Salgaller M, Agius L, Yates A, Pearl D, Roberts W and Stephens R (1990) Application of automated image analysis to demonstrate correlation between

© Cancer Research Campaign 1997

Ras p21 expression and severity of gliomas. *Biochem Biophysical Res Comm* **169**: 482–491

Scheithauer BW (1990) Tumors of the meninges: proposed modifications of the World Health Organization classification. *Acta Neuropathol* **80**: 343–354

Stacey DW, Feig LA and Gibbs JB (1991) Dominant inhibitory Ras mutants inhibit the activity of either cellular or oncogenic Ras. *Mol Cell Biol* **11**: 4053–4064

Stahle-Backdhal M, Inoue M, Zedenius J, Sandstedt B, Demarco L, Flam F, Silfersward C, Andrade J and Freidman E (1995) Decreased expression of Ras GTPase activating protein in human trophoblastic tumors. *Am J Pathol* **146**: 1073–1078

Takahashi K, Suzuki H, Hatori M, Abe Y, Kokubun S, Sakurai M and Shibahara S (1995) Reduced expression of neurofibromin in the soft tissue tumours obtained from patients with neurofibromatosis type 1. *Clin Sci* **88**: 581–585

Tikoo A, Varga M, Ramesh V, Gusella J and Maruta H (1994) An anti-Ras function of neurofibromatosis type 2 gene product (NF2/merlin). *J Biol Chem* **269**: 23387–23390

Todo T, Adams EF, Fahlbush R, Dingermann T and Werner H (1996) Autocrine growth stimulation of human meningioma cells by platelet-derived growth factor. *J Neurosurg* **84**: 852–859

Trahey M and McCormick F (1987) A cytoplasmic protein stimulates normal N-ras p21 GTPase, but does not affect oncogenic mutants. *Science* **238**: 542–545

Trofatter JA, MacCollin MM, Rutter JL, Murrell JR, Duyao MP, Parry DM, Eldridge R, Kley N, Menon AG, Pulaski K, Haase VH, Ambrose CM, Munroe D, Bove C, Haines JL, Martuza RL, Macdonald ME, Seizinger BR, Short MP, Buckler AJ and Gusella JF (1993) A novel meosin-, ezrin-, radixin-like gene is a candidate for the neurofibromatosis 2 tumor suppressor. *Cell* **72**: 791–800

Vogel KS, Brannan CI, Jenkins NA, Copeland NG and Parada LF (1995) Loss of neurofibromin results in neurotrophin-independent survival of embryonic sensory and sympathetic neurons. *Cell* **82**: 733–742

Von Deimling A, Krone W and Menon GA (1995) Neurofibromatosis type 1: pathology, clinical features and molecular genetics. *Brain Pathol* **5**: 153–162

Weisbach L, Settleman J, Kalady MF, Snijders AJ, Murthy AE, Yan Y-X and Bernards A (1994) Identification of a human RasGAP-related protein containing calmodulin-binding motifs. *J Biol Chem* **269**: 20517–20521

Xu G, Lin B, Tanaka K, Dunn D, Wood D, Gesteland R, White R, Weiss R and Tamanoi F (1990) The catalytic domain of the neurofibromatosis type 1 gene product stimulates ras GTPase and complements *ira* mutants of *S. cerevisiae*. *Cell* **63**: 835–841

Yamada K, Kondo T, Yoshioka M and Oami H (1980) Cytogenetic studies in twenty human brain tumors: association with No. 22 abnormalities with tumours of the brain. *Cancer Genet Cytogenet* **2**: 293–307

*© Cancer Research Campaign 1997*